# Determinants of early marriage among married women in Injibara town, north West Ethiopia: community-based cross-sectional study

**DOI:** 10.1186/s12905-019-0832-0

**Published:** 2019-11-08

**Authors:** Minale Bezie, Dagne Addisu

**Affiliations:** Department of midwifery, college of health science, Debre Tabor University, Debre Tabor, Ethiopia

**Keywords:** Early marriage, Prevalence, Ethiopia, Determinant factors

## Abstract

**Background:**

Early marriage is occurred when one or both of the spouses are below the age of 18 years at the time of their first marriage. It is one of the major traditional practices in developing counties particularly in Ethiopia; which has significant physical, intellectual, psychological and emotional effects and reduces educational opportunities and the chance for personal growth for both boys and girls. Even though this traditional practice was the common cultural events in the study area, there is no prior study on the magnitude and its determinant factors. Hence, the study was aimed to determine the prevalence and determinant factors of early marriage among married women in Injibara town, North West Ethiopia.

**Methods:**

A Community-based cross-sectional study was conducted from September to December 2018. A total of 373 women were included in the study. A multistage sampling procedure was applied to select the study participants. Data analysis was done by using SPSS versions 23. Both descriptive & analytical statistics were computed. Statistical significance was considered at *P* < 0.05 and the strength of association were assessed by using adjusted odds ratio with 95% confidence interval.

**Result:**

The prevalence of early marriage was 167(44.8%). The minimum and maximum ages at first marriage were 9 and 23 years respectively. Non-formal educational level of the father [Adjusted Odd Ratio (AOR) =2.32; 95%CI = 1.33–4.05], family’s average monthly income <1000 Ethiopian birr [AOR = 2.32, 95%CI = 1.27–4.24], family size ≥7 [AOR = 3.59, 95%CI = 1.94–6.63] and non-formal education level of the respondents [AOR = 5.16; 95%CI = 2.87–9.28] were found to be associated with early marriage.

**Conclusion:**

The prevalence of early marriage was high in Injibara town, Ethiopia. Factors that tend to facilitate early marriage in this town include family income, family size, educational level of the father and that of the respondent. Improving on the strategies that promote formal education will reduce the level of early marriage in Injibara town, Ethiopia.

## Background

Early marriage is defined as the marriage of children and adolescents below the age of 18 years when the girl is not yet physically and emotionally mature enough to bear a child and take the social responsibility of the wife. Recently more than 60 million child girls and women were affected by early marriage globally [1, 2].

Sub-Saharan Africa had the highest rates of early child marriage in the world. From 20 countries that had the highest rate of girl child marriage worldwide, 18 were found in the Sub-Saharan region. Evidence also reported that more than half of the girls in the region marry before 18 years in many countries in the sub-Saharan region [3, 4].

Child marriage and harmful traditional practices are the most common socio-cultural events in most rural areas of Ethiopia. According to the Ethiopian demography and health survey (EDHS) 2016, the national prevalence of early marriage was 58%. The magnitude of early marriage also 87 and 80% in East Gojjam and South Wollo zone of Amhara region, Ethiopia respectively [5–8].

Early child marriage practices were a significant social concern globally in recent years due to dangerous health consequences such as increased risk of acquiring sexually transmitted diseases, child malnutrition, teenage pregnancy, miss the opportunity of formal education, dropping out of school and maternal and child morbidity and mortality on young women who marry at early ages [9, 10].

In Ethiopia, early girl marriage has significant health and socioeconomic impacts on married women. Some of these consequences include adverse pregnancy outcomes, miss the chance of formal education, lack of opportunity for salary employment and social power inequities, such as sexual violence, imbalanced profit-producing opportunity, little money for achieving their regular necessities and gender inequality in and out of their households [11].

Although the concerns of child marriage and the well-being of Ethiopian girls have received increased attention on recent years, most females in Injibara town still enter marriage at much younger ages and early marriage is a common harmful traditional practice in the town. However, the magnitude and its determinant factors are unknown. Therefore, this study was aimed to determine the prevalence and determinant factors of early marriage among married women in Injibara town, North West Ethiopia. This finding is important to evaluate the effectiveness of efforts done previously to eliminate child marriage and to design evidence-based intervention for the reduction of child marriage. It may also useful to improve laws against early marriage, designing and targeting adolescent health and human rights strategies.

## Methods

### Study area and period

The study was conducted in Injibara town, Amhara region, Ethiopia from September to December 2018. Injibara is the administrative center of the Awi Zone in the Amhara Region, which is located 447 kms from the capital city of Addis Ababa.

The legal age of marriage is 18 years and above in Ethiopia. However, several girls go into marriage at younger ages in Injibara town. In the study area, marriages are usually organized by the families and heads, and heads from the husband’s family will communicate the spouse’s family and bid moneys as bride value. In addition, marriage also practiced through abducting girls and then sending community leaders and elders to the female’s family for negotiation and to give bride price. During this time, the female’s family will usually decide to accept the marriage and the provided bride price. However, harmful traditional practices such as rapping and abducting women are prohibited in the country.

### Study design

Community-based cross-sectional study design was used.

### Study population

All married women in Injibara Town were the source population. This study included all married women aged 15–49 years and who lived at selected kebeles in Injibara town. Those married women aged 15–49 years and who did not stay more than 6 months in the town was excluded.

### Sample size determination

The sample size was determined using a single population proportion formula by assuming a 95%CI, 4% marginal of error, and the prevalence of early marriage 83% [8]. After 10% of the non-response rate was added; the final sample size for this study was 373.

### Sampling technique and procedure

A multistage sampling procedure was adopted from previously published researches [8, 12] and employed to select study participants. Injibara town has five kebeles (the lowest administrative unit in the government administrative structure) and three kebeles out of five were selected by a simple random sampling technique with the lottery method. The selected kebeles were then subdivided into village/Got. Lists of the households in each village were obtained from the kebele administrative offices. First households were allocated proportionally in each selected village and then households were selected by simple random sampling method from each village by using the number of the household as a sampling frame. The first households were selected from the town using the town’s house number registration by lottery method. In cases of selected households with more than one eligible study subject, only a single respondent was chosen by the lottery method. In cases where no candidate respondent was found in the selected household, the data collectors have gone to the next household until they found an eligible study subject.

### Data collection instruments and technique

Data was collected using a structured interviewer administered questionnaires. The questionnaires were adapted and modified from previous similar literature after considering the local situations [9, 11, 13, 14]. Before pretest, the questionnaires were sent to two experts for evaluation and then some modification was made on the tool after receiving their suggestion. The questionnaires were designed first in English then translated to the local language Amharic by a language expert for data collection and transcribed back to English to check for consistency. Before the actual data collection period; the questionnaires were pretested on 5% of married women in Bahir dar town, thereby adjustment was made on the tool. Data was collected by four nurses and four health extension workers. Two- day training was given on the objective of the study, clarity of the tool and technique and time of interview for data collectors and supervisor prior to data collection. Respondents were asked their age retrospectively at which their marriage agreement was made between them and their first spouse.

### Data management and analysis

Data were checked for completeness and inconsistencies. Then the data were coded and entered into EPI data version 3.1 then exported to SPSS version 23 for analysis. Descriptive and analytic statistics were computed.

Variables with *p*-values ≤0.2 in bivariate analysis remained in the model as potential confounders for the next level analysis. In multivariable logistic regression; statistical significance was considered at *P* < 0.05. Adjusted odd ratio (AOR) with 95% confidence interval (CI) was used to measure the strength of association between early marriage and predictor variables. The backward stepwise logistic regression method was used in multiple logistic regressions.

## Results

### Socio-demographic characteristics of mothers

In this study, a total of 373 married women were interviewed. The overall response rate was 100%. Among the total respondents, 167 (44.8%) of respondents had first marriage below 18 years. The minimum and the maximum age at first marriage were 9 and 23 years with the mean age of 17 years with standard deviation of ±3.2 years (Table 1).

**Table 1:** Socio-demographic characteristic of married women in Injibara town, Awi Zone, North West Ethiopia, 2018 (*N* = 373)

Variable	Frequency	Percent (%)
Age at first marriage		
< 18 years	167	44.8
≥ 18 years	206	55.2
Current age (in a year)		
≤ 20	62	16.6
21–30	246	66
> 30	65	17.4
Religion		
Orthodox	305	81.8
Others (Muslim & protestant)	68	18.2
Ethnicity		
Agew	309	82.8
Others (Amhara & gumez)	64	17.2
Father educational status		
Non-formal	159	42.6
Formal	214	57.4
Mother educational status		
Non-formal education	268	71.8
Formal education	105	28.2
Husband educational status		
Non-formal	144	38.6
Formal	229	61.4
Educational status of respondents		
Non-formal	267	71.6
Formal	106	28.4
Occupation		
Housewife	139	37.3
Civil servant	95	25.5
Merchant	139	37.3
Family income		
< 1000	133	35.7
1000–200	132	35.4
> 2000	108	29
Family size		
1–3	124	33.2
4–6	138	37
≥ 7	111	29.8

### Marriage related characteristics of respondents

All of the respondents (100%) received a bridge price for their marriage and around 15.2% of the respondents left from school for marriage. The majority, 295(79.1%) of respondents didn’t gave their consent at their first marriage (Table 2**).**

**Table 2:** Marriage related characteristics married women in Injibara town, Awi Zone, North West Ethiopia, 2018 (*N* = 373)

Variables	Frequency	Percent (%)
Made to leave school to marry		
Yes	59	15.2
No	314	84.2
Marriage arrangement at first marriage		
Arranged by others	333	89.3
Chosen	25	6.7
Abducted	15	4
Decision-makers for girl marriage		
Fathers only	95	25.5
Both parents	197	52.8
Community leaders	23	6.2
Religious leaders	24	6.4
Gave consent at their first marriage		
Yes	78	20.9
No	295	79.1

### Reasons for early marriage

Increase bonding between two families (74%), material benefits (20.4%), unable to cover to educate all children (3.8%) and ensuring virginity up to marriage (1.8%) were reasons for early girl marriage in Injibara town.

### Determinants of early marriage

In the bivariable analysis, family size, ethnicity, the education status of the father, educational status of the husband, educational status of respondent and family income became significant at the level of 0.2.

However, non-formal educational level of the father [Adjusted Odd Ratio (AOR) =2.32; 95%CI = 1.33–4.05], family’s average monthly income <1000 Ethiopian birr [AOR = 2.32; 95%CI = 1.27–4.24], family size ≥7 [AOR = 3.59; 95%CI = 1.94–6.63] and non-formal education level of the respondents [AOR = 5.16; 95%CI = 2.87–9.28] were found to be associated with early marriage in multivariable logistic regression (Table 3).

**Table 3:** Bivariable and multivariable associations of early marriage and independent factors among married women in Injibara Town, North West Ethiopia, 2018

Variables	Age at marriage		Crude Odd Ratio (COR) (95%CI)	Adjusted Odd Ratio (AOR) (95%CI)
	<18 year	≥18 year		
Ethnicity				
Agew	142 (46.7%)	162 (53.3%)	1.54 (0.89–2.64	1.06 (0.56–1.99)
Others	25 (36.2%)	44 (63.8%)	1	1
Father education				
No formal education	101 (63.5%)	58 (36.5%)	3.90 (2.53–6.02)	2.34 (1.42–3.85)**
Formal education	66 (30.8%)	148 (69.2%)	1	1
Husband education				
No formal education	92 (63.9%)	52 (36.1%)	3.63 (2.34–5.62)	1.55 (0.87–2.76)
Formal education	75 (32.8%)	154 (67.2%)	1	1
Educational status of respondents				
No formal education	144 (53.9%)	123 (46.1%)	4.22 (2.51–7.11)	3.94 (2.22–7.00)**
Formal education	23 (21.7%)	83 (78.3%)	1	1
Family income (birr)				
< 1000	82 (61.7%)	51 (38.3%)	3.49 (2.04–5.98)	2.85 (1.54–5.26)**
1000–2000	51 (38.6%)	81 (61.4%)	1.37 (0.80–2.34)	1.27 (0.70–2.33)
> 2000	34 (31.5%)	74 (68.5%)	1	1
Family size				
1–3	39 (31.5%)	85 (68.5%)	1	1
4–6	53 (38.4%)	85 (61.6%)	1.35 (0.81–2.26)	1.26 (0.72–2.22)
≥ 7	75 (67.6%)	36 (32.4%)	4.54 (2.62–7.86)	3.59 (1.94–6.63)**

** mean *p*-value < 0.05

Others mean Amhara and Gumez

## Discussion

The prevalence of early marriage in Injibara town was 44.8% with [95%CI = 39.5–49.9]. This finding was lower than a study finding in Bangladeshi (78.2%) [15]. This event could be due to the difference in the implementation of regulatory and legislative measures, cultural, traditional, religious and social norms and values between the study areas.

This finding was also lower than a study conducted in Sub-Saharan Africa (55%) [16] and East Gojjam Zones, Amhara region (83%) [8]. The difference might be due to this study was conducted more recently in which concerns of child marriage, and the well-being of females have received increased attention, and the level of awareness for harmful traditional practice has improved. Furthermore, ethical and cultural variation between study areas and drawing policy actions and adopting legislative fireworks to reduce early marriage may also contribute to this difference.

This finding was higher than a study conducted in Latin America (16%) [17]. This difference could be due to the former study was conducted in developed countries in which the best child marriage ages and the effects of early marriage were well comprehended, and children’s reproductive rights were properly utilized. Besides, marriages in the current study were conducted through arrangements by others (parents) and through abducting girls which may increase the magnitude of early marriage.

The present findings revealed that educational level of the respondents [AOR =3.94, 95%CI = 2.22–7.00] and educational level of the fathers [AOR =3.94, 95%CI = 2.22–7.00] had a significant association with girl early marriage. Females with non-formal educational status had 3.94 times higher odds of having an early marriage as compared to their counterparts. This finding was in agreement with the study finding from the Democratic Republic of Congo [18], Serbia [9] and Sudan [19]. The higher one’s educational attainment, the more knowledge he/she gets and understands, including all information about reproductive health, the best marriage age, and the effect of having an early marriage. But if one’s educational achievement is low, there will be a disconnection of knowledge and information and also fewer youth activities. Moreover, the role of parents in the continuity of early marriage is mainly inseparable from their knowledge linked to their educational achievement. Parents with less understanding of family life may consider early marriage as the best solution to create a better relationship with others [20].

In this study, females with average family income <1000 Ethiopian birr were 2.85 times more likely to practice early girl marriage as compared with those who had >2000 birrs [AOR =2.85, 95%CI = 1.54–5.26]. This might be because of poverty. Poverty is signified as both a cause and a consequence of early marriage which makes parents marry off their children at a younger age. Parents think that girls are an economic burden. As a result, they try to retain their economic circumstances by division of roles and responsibilities from the girl’s family to the husband [20].

The present study also found that family sizes ≥7 was significantly associated with early girl marriage [AOR = 3.59, 95%CI = 1.94–6.63]. The odd of early marriage were 3.59 times higher among family size greater or equal to seven as compared to those who had less than or equal to three. This could be due to a large family size that might lead to the greater dilution of parental resources. Hence, parents may prefer early girl marriage to decrease the dilution of parental resources and to improve the family’s economy by receiving bridge prices for girl marriage.

We used a small sample size that may affect the generalization to the target population. Besides, as the study was conducted in a single town, the results might not be representative of the country. Furthermore, respondents may not remember their exact age at their first marriage because of memory lapses and lack of a vital registration system. As a result, this study may be susceptible to recall bias.

## Conclusion

The prevalence of early marriage was high in Injibara town, Ethiopia. Factors that tend to facilitate an early marriage in this town include family income, family size, the educational level of the father and the respondent. Improving on the strategies which promote formal education will reduce the level of early marriage in Injibara town, Ethiopia. Besides, improve family income will also reduce the level of early marriage in Injibara town, Ethiopia.

## Data Availability

The datasets used in this study are available from the corresponding author on reasonable request.

## Abbreviation

AOR: Adjusted odd ration
COR: Crude odd ratio
FDRE: Federal Democratic of Ethiopia
FMRW: Forum on Marriage and Rights of Women’s
HEW: Health Extension Workers
ICF: International Coach Federation
NCTPE: Natural Committee on Traditional Practice of Ethiopia
NGO: Non-governmental Organization
